# MiR-181d-5p Targets KLF6 to Improve Ischemia/Reperfusion-Induced AKI Through Effects on Renal Function, Apoptosis, and Inflammation

**DOI:** 10.3389/fphys.2020.00510

**Published:** 2020-05-27

**Authors:** Yue Zhang, Chenyu Li, Chen Guan, Bin Zhou, Lin Wang, Chengyu Yang, Li Zhen, Jie Dai, Long Zhao, Wei Jiang, Yan Xu

**Affiliations:** ^1^Department of Nephrology, The Affiliated Hospital of Qingdao University, Qingdao, China; ^2^Nephrologisches Zentrum, Ludwig Maximilian University of Munich, Munich, Germany

**Keywords:** MiR-181d-5p, KLF6, IRI, inflammation, apoptosis, renal function

## Abstract

Renal tubular epithelial cell (RTEC) death and renal interstitial inflammation are the most crucial pathophysiological changes in acute kidney ischemia/reperfusion injury (IRI). The microRNA (miR)-181d family plays diverse roles in cell proliferation, apoptosis and inflammation, but its renal target and potential role in IRI are unknown. Here, we showed that the expression of miR-181d-5p decreased and Krueppel-like factor 6 (KLF6) increased in a renal cell (HK-2) model of hypoxia/reoxygenation (H/R) injury and a mouse model of renal IRI. They were mainly distributed in the renal tubules. After renal IRI, miR-181d-5p overexpression significantly inhibited inflammatory mediators, reduced apoptosis and further improved renal function. KLF6 exacerbated RTEC damage and acted as a NF-κB co-activator to aggravate the renal IRI inflammatory response. Mechanistically, KLF6 was predicted as a new potential target gene of miR-181d-5p through bioinformatic analysis and luciferase reporter assay verification. After overexpressing miR-181d-5p and inhibiting KLF6, the role of miR-181d-5p was weakened on the renal damage improvement. In conclusion, miR-181d-5p upregulation produced protective antiapoptotic and anti-inflammatory effects against IRI in kidneys *in vivo* and H/R injury in HK-2 cells *in vitro*, and these effects were achieved by targeted inhibition of KLF6. Thus, our results provide novel insights into the molecular mechanisms associated with IRI and a potential novel therapeutic target.

## Introduction

Ischemia/reperfusion injury (IRI) is the outcome of an inflammatory process and tubular cell death triggered by a transient reduction in or cessation of blood flow followed by reperfusion (Lameire et al., 2005). Unresolved IRI can contribute to chronic kidney disease and even death. Numerous studies have investigated the molecular and cellular mechanisms of renal IRI, suggesting the occurrence of various pathophysiological changes, including tubular epithelial cell injury, microvascular dysfunction, and inflammation, which show dramatic contributions to overall renal damage in IRI (Schiffl, 2002; Lopez-Neblina et al., 2005; Bonventre and Yang, 2011; Jang and Rabb, 2015; Wu et al., 2018). However, the underlying mechanism has not been fully elucidated, and no effective therapies are available for I/R-associated kidney injury.

In the past decade, numerous studies have focused on studying the role of miRNAs, including miR-126 (Bijkerk et al., 2014a, b), miR-494 (Lan et al., 2012), miR-21 (Hu et al., 2014), miR-687 (Bhatt et al., 2015), miR-150 (Ranganathan et al., 2015), and miR-489 (Wei et al., 2016), in prognosis and therapeutic strategies for IRI-related kidney diseases (Baker et al., 2017).

The microRNA (miR)-181 family plays diverse roles in regulating key aspects of cellular growth, development, and activation. Accumulating evidence supports an important role for the miR-181 family in inflammation via the control of critical signaling pathways, such as downstream NF-κB signaling (Sun et al., 2012; Cao et al., 2017), and targets relevant to immune cell homeostasis (Xue et al., 2011; Seeger et al., 2013). A recent study showed that miR-181d activated the NF-κB pathway and promoted the expression of the proinflammatory cytokines TNF-α and IL-12. Inhibition of the NF-κB pathway suppressed DC maturation (Su et al., 2017). Upregulation of miR-30a and miR-181d was related to increased IL-6 levels via the modulation of SOCS3 expression, which increased the survival rate of mice (Fan et al., 2019). Furthermore, miR-181d was found to mediate tumorigenesis and tumor development as a tumor suppressor. Ectopic expression of miR-181d suppressed cell proliferation and promoted apoptosis in osteosarcoma, colorectal cancer, gastric cancer, human esophageal squamous cell carcinoma and glioma (Wang et al., 2012; Li et al., 2016; Guo et al., 2017; Chen et al., 2018; Huang et al., 2018; Jiang et al., 2019). The expression levels of 76 miRNAs in the kidneys of mice with IRI were changed by at least two-fold, and the expression level of miR-181d was significantly decreased (Chen et al., 2004; Liu et al., 2012). However, whether miR-181d plays an important role in the regulation of renal IRI is unknown.

KLF6 is a unique member of the zinc finger family of transcription factors (De Graeve et al., 2003). KLF6 regulates the expression of genes involved in signal transduction; cell proliferation, differentiation, and death; oncogenesis; and inflammation. Some studies have demonstrated that KLF6 promotes proinflammatory gene expression while suppressing anti-inflammatory gene expression in macrophages (Date et al., 2014; Kim et al., 2016, 2020). Furthermore, in intestinal inflammation, KLF6 promotes proinflammatory gene expression through the enhancement of NF-κB signaling while simultaneously suppressing anti-inflammatory gene expression through the repression of STAT3 signaling (Date et al., 2014; Goodman et al., 2016; Kim et al., 2016). Moreover, a previous study identified the KLF6 gene as a tumor suppressor gene; for example, KLF6 inhibited the proliferation and promoted the apoptosis of clear cell renal cell carcinoma (ccRCC) cells as a target gene of both miR-543 and miR-181a (Gao et al., 2017; Lei et al., 2018; Yang et al., 2018). Furthermore, in both normal and tumor contexts, KLF6 is required for oxidative and oncogene-induced cellular senescence (Sabatino et al., 2019). KLF6 maintains mitochondrial function and prevents activation of intrinsic apoptotic pathways in injured renal podocytes through transcriptional regulation of synthesis of cytochrome c oxidase (SCO2) protein expression (Mallipattu et al., 2015). Differential gene expression analysis showed that KLF6 exhibited a significant difference in expression in the early stage of IRI, suggesting that it was strongly associated with IRI. However, KLF6 gene regulation has not been extensively explored in the IRI model.

Our previous bioinformatic study found that the downstream target gene of miR-181d-5p is KLF6, which is closely related to IRI. In this study, we established a H/R HK-2 cell model and a mouse I/R model to examine the role and regulation of miR-181d-5p in renal IRI and to identify the underlying signal transduction pathways. We verified the induction of miR-181d-5p expression during renal IRI and found that this miRNA may be targeted to suppress KLF6 expression in order to protect kidney cells and tissues against injury.

## Results

### Expression and Localization of miR-181d-5p During Renal IRI *in vivo* and Hypoxia *in vitro*

**FIGURE 1:**
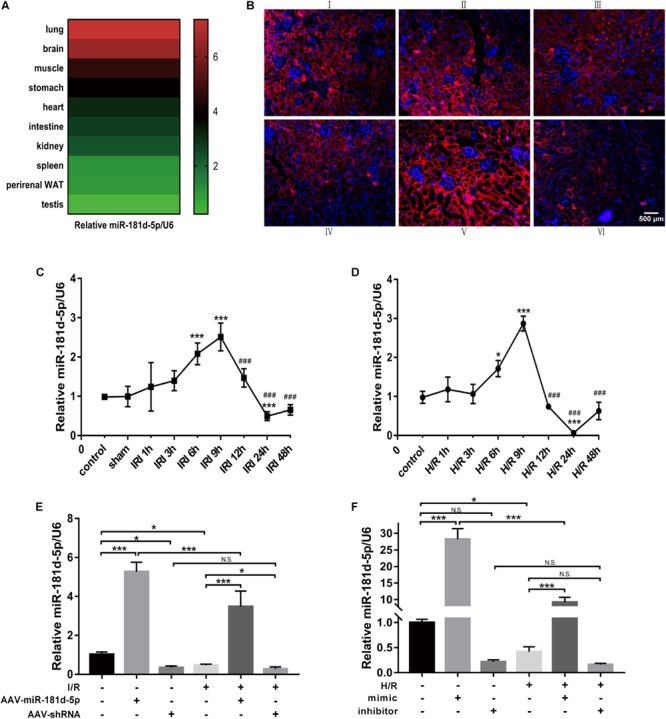
Expression and localization of miR-181d-5p during renal IRI *in vivo* and hypoxia *in vitro*. **(A)** Total RNA samples were extracted from various tissues of C57BL/6 mice. MiRNAs were reverse transcribed using miR-181d-5p and U6 RNA-specific primers, and qRT-PCR was performed as described in the section “Materials and Methods.” The relative expression of mature miR-181d-5p was normalized to that of U6 RNA (*n* = 4 per group). **(B)** The localization of miR-181d-5p in the mouse kidney after IRI (45 min of bilateral renal ischemia followed by 24 h of reperfusion) was assessed by *in situ* hybridization. Paraffin-fixed mouse kidney sections were hybridized with the digoxigenin-labeled miR-181d-5p fluorescent probe (red), and nuclei were stained with DAP1 (blue). Scale bar, 500 μm. (I: control; II: sham; III: I/R; IV: I/R+AAV-control; V: I/R+AAV-miR-181d-5p; VI: IRI+AAV-shRNA.) **(C)** Time course of the miR-1R1d-5p expression levels in mouse kidneys. Tissues were harvested at different time points after the bilateral renal pedicle was clamped for 45 min (*n* = 4 or 5 per group). **(D)** Time course of the miR-181d-5p expression levels *in vitro*. MiR-181d-5p induction was assessed in cultured HK-2 cells subjected to H/R. HK-2 cells were incubated under normoxia or hypoxia (1% oxygen) for 0–48 h and subsequently reoxygenated for 3 h. Total RNA samples were extracted for RT-PCR, showing significant induction of miR-181d-5p after treatment with hypoxia for 24 h/reoxygenation for 3 h (*n* = 5 per group). **(E,F)** Quantitative analysis of miR-181d-5p expression in mice kidneys and HK-2 cells treated with or without miR-181d-5p AAV constructs (*n* = 5 per group) The data are presented as the means ± SDs. ^∗^ and #, *P* < 0.05; ^∗∗^ and *##, P* < 0.01; ^∗∗∗^ and ###, *P* < 0.001.

We used quantitative real-time PCR (qRT-PCR) to investigate miR-181d-5p expression under normal physiological conditions and found that it was highly expressed in the brain and lung; moderately expressed in the muscles, stomach, intestine, hearts and kidneys; and expressed at the lowest levels in the spleen, liver, testis, and perirenal white adipose tissue (Figure 1A). Renal IRI was induced in mice via bilateral renal artery occlusion for 45 min and reperfusion for the indicated time. qRT-PCR showed that miR-181d-5p levels increased gradually in the IRI group, peaking at 9 h but declining at 24 h compared with those in the normal control and sham groups (Figure 1C). Interestingly, the *in situ* hybridization assay results revealed that miR-181d-5p staining was distributed mainly in renal tubular epithelial cells (RTECs) in the renal cortex (Figure 1B). Furthermore, HK-2 cells were cultured under hypoxia (5% CO_2_, 1% O_2_, and 94% N_2_) for the indicated time followed by reoxygenation for 3 h. Compared with the miR-181d-5p level in the non-hypoxia-exposed groups, the miR-181d-5p in the H/R group increased at 6–12 h, but started to decrease at 24 h (Figure 1D). MiR-181d-5p expression was increased in mice kidneys and HK-2 cells overexpressing miR-181d-5p, but it exhibited a decreasing but non-significant trend after inhibitor transfection in HK-2 cells. Regardless of whether miR-181d-5p was overexpressed or suppressed, its expression exhibited a downward trend after either I/R or H/R (Figures 1E,F).

### MiR-181d-5p Improves Kidney Function in Mice With Renal IRI and Ameliorates H/R-Induced Damage in H/R-Exposed HK-2 Cells

**FIGURE 2:**
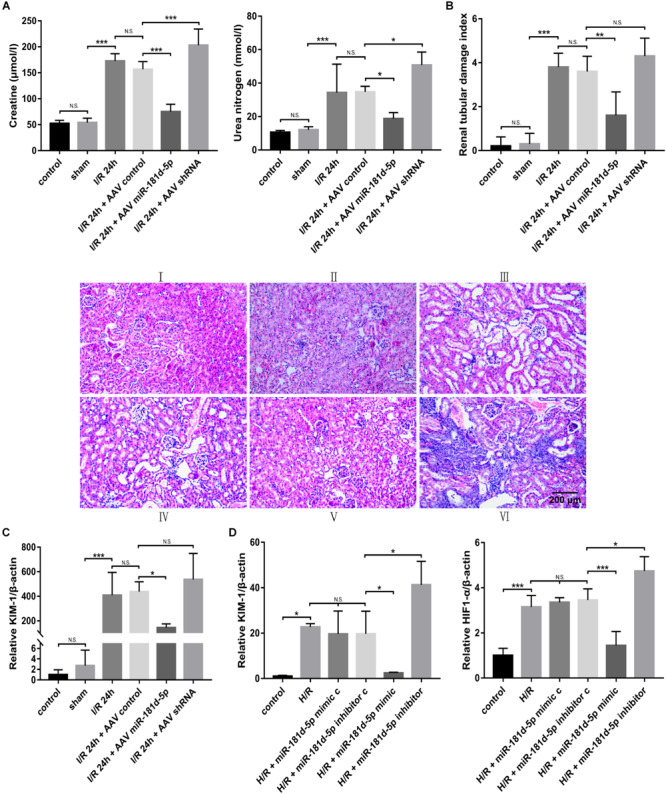
MiR-181d-5p improved kidney function in mice with renal IRI and in RTECs. C57BL/6 mice were injected with AAVs and, 3 weeks later, were subjected to 45 min of bilateral renal ischemia followed by 24 h of reperfusion. HK-2 cells were transfected with 100 nM miR-181d-5p mimic, 150 nM miR-181d-5p inhibitor or scrambled oligonucleotides and, 72 h later, were incubated in normoxia (control) or treated with hypoxia (1% oxygen) for 24 h/reoxygenation for 3 h. **(A)** Serum creatinine and BUN levels were assessed in mice with or without miR-181d-5p infusion after IRI (*n* = 6 per group). **(B)** Pathological score of renal tubular injury in mice infused with or without miR-181d-5p, as assessed using hematoxylin and eosin staining. Paraffin sections were stained with hematoxylin and eosin. Scale bar, 200 μm (I: control; II: sham; III: I/R; IV: I/R+AAV-control; V: I/R+AAV-miR-1818d-5p; VI: IRI+AAV-shRNA.) **(C)** qRT-PCR was performed for KIM-1 as described in the section “Materials and Methods.” The relative expression of KIM-1 was normalized to that of β-actin (*n* = 5 per group). **(D)** The relative mRNA expression levels of KIM-1 and HIF-1α in HK-2 cells were normalized to the β-actin expression level (*n* = 5 per group). The data are presented as the means ± SDs. *P < 0.05, **P < 0.01, ***P < 0.001.

Control adeno-associated virus (AAV-control), AAV-miR-181d-5p or AAV-shRNA was perfused into the bilateral kidney parenchyma of mice before treatment with ischemia for 45 min and reperfusion for 24 h. Compared to mice in the control and sham groups, mice in the IRI group exhibited elevated serum creatinine (Cr) and blood urea nitrogen (BUN) levels, and mice treated with miR-181d-5p showed significantly lower serum creatinine and BUN levels (Figure 2A). We further examined renal pathological injury by hematoxylin and eosin (HE) staining. Figure 2B showed that kidneys from sham group mice exhibited normal histology, but those from IRI and AAV-control group mice exhibited severe kidney injury with tubular damage. However, AAV-miR-181d-5p injection significantly reduced tubular damage to approximately half that observed in AAV-control group mice. Furthermore, the level of kidney injury molecule-1 (KIM-1) in renal cortical tissues was determined by qRT-PCR and was found to be significantly decreased after miR-181d-5p overexpression (Figure 2C). These results suggested that miR-181d-5p may play a protective role against renal IRI in mice. Similarly, we examined the effect of miR-181d-5p on HK-2 cell injury induced by 24 h of hypoxia/3 h of reoxygenation. H/R treatment led to a significant increase in hypoxia-inducible factor 1α (HIF-1α) and KIM-1 expression in HK-2 cells transfected with the miR-181d-5p control. Transfection with the miR-181d-5p mimic and inhibitor generally increased and decreased the levels of these molecules, respectively (Figure 2D). These *in vitro* results suggest that miR-181d-5p may act as a protective factor against tubular cell H/R injury.

### MiR-181d-5p Reduces Apoptosis After I/R or H/R

**FIGURE 3:**
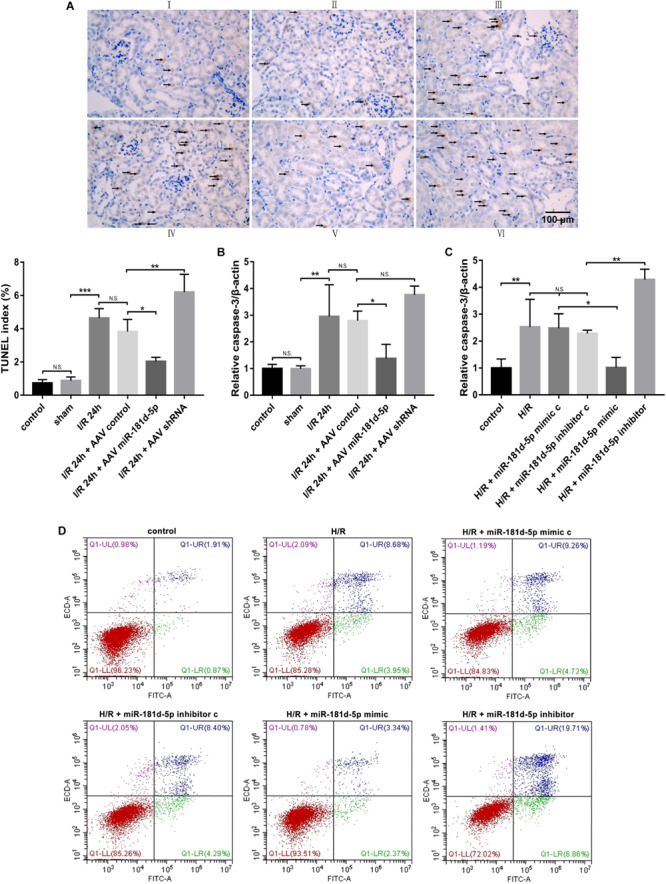
MiR-181d-5p decreased RTEC apoptosis during IRI in mice and H/R in HK-2 cells. **(A)** Apoptotic kidney cells in mice infused with or without miR-181d-5p, as assessed using TUNEL. TUNEL of representative kidney sections from each experimental group is shown. Colocalization of blue and brown staining in nuclei indicates apoptotic cells, which are indicated with arrows. Scale bar. 100 mm. (I: control; II: sham; III: I/R; IV: I/R+AAV-control; V: I/R+AAV-miR-181d-5p; VI: IRI+AAV-shRNA). Proportions of TUNEL-positive nuclei to total nuclei in renal epithelial cells of mice are shown (*n* = 3 per group). **(B,C)** qRT-PCR was used to detect caspase-3 in mouse tissues and HK-2 cells as described in the section “Materials and Methods.” The relative expression of caspase-3 was normalized to that of β-actin (*n* = 5 per group). **(D)** Flow cytometric analysis of Annexin V/PI staining showed that hsa-miR-181d-5p decreased the level of programmed cell apoptosis in miR-181d-5p-treated HK2 cells. The data are presented as the means-SDs. *P < 0.05, **P < 0.01, ***P < 0.001.

Necrosis and apoptosis of RTECs caused by renal IRI are major causes of acute renal failure, and accumulating evidence has indicated that IRI-induced cell death is associated with apoptosis (Wu et al., 2018). We examined RTEC apoptosis using terminal deoxynucleotidyl transferase-mediated deoxyuridine nick-end labeling (TUNEL) with digoxigenin-labeled deoxyuridine triphosphate in the previously described groups of mice. Compared to the control and sham groups, the IRI group exhibited an increased degree of RTEC apoptosis. MiR-181d-5p overexpression resulted in a significant decrease in apoptosis compared with that in the IRI group (Figure 3A). Similarly, *in vitro*, Annexin V-FITC/propidium iodide (PI) double staining confirmed obvious cell apoptosis after miR-181d-5p transfection (Figure 3D). In addition, the level of caspase-3, a marker of apoptosis, was significantly reduced in the renal injury group and decreased in the miR-181d-5p overexpression group both *in vivo* and *in vitro* (Figures 3B,C). These data suggest that miR-181d-5p may play important roles in cell death during renal IRI.

### MiR-181d-5p Reduces I/R- or H/R-Induced Inflammatory Responses

**FIGURE 4:**
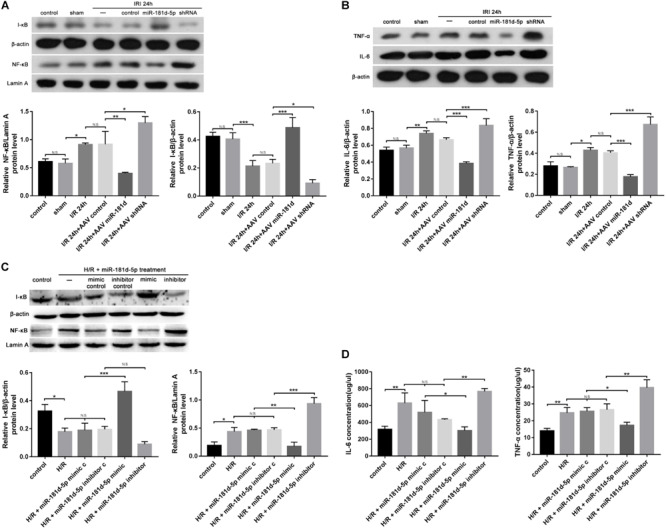
MiR-181d-5p reduced inflammation-related gene expression in mice with IRI and HK-2 cells subjected to H/R. **(A,C)** MiR-181d-5p decreased NF-KB expression and increased I-KB expression in HK-2 cells subjected to H/R and mice subjected to I/R that were transfected with miR-181d-5p. The protein expression are shown from Western blot analysis. β-Actin and Lamin-A were used as internal controls for I-KB and NF-KB, respectively (*n* = 3 per group). **(B)** Western blot analysis of IL-6 and TNF-α in damaged kidneys of mice treated with different miR-181d-5p constructs (*n* = 3 per group). **(D)** ELISAs were used to measure IL-6 and TNF-α levels in the cell supernatant. The data are presented as the means ± SDs (*n* = 4 or 5 per group). *P < 0.05, **P < 0.01, ***P < 0.001.

Postischemic NF-kB activation in renal tubular epithelia aggravates kidney injury and exacerbates a maladaptive inflammatory response (Barnes, 1997; Markó et al., 2016). Nuclear and cytosolic extracts were probed with anti-NF-κB and anti-I-κB antibodies, respectively, to quantify protein levels. The NF-κB mRNA and protein levels in nuclear extracts from mouse renal cortexes infected with AAV containing miR-181d-5p were markedly decreased compared with those in mice not treated with miR-181d-5p; conversely, the mRNA and protein levels of I-κB were increased (Figure 4A). As shown in Figure 4C, we verified that NF-κB was induced and activated by treatment with hypoxia for 24 h/reoxygenation for 3 h in HK-2 cells and was decreased by treatment with miR-181d-5p. The Western blot analysis results showed that overexpression of miR-181d-5p elevated IL-6 and TNF-α mRNA and protein levels in mice after I/R compared with those in the control and sham groups. Similarly, the renal IRI-induced release of inflammatory mediators (IL-6 and TNF-α levels) was decreased with miR-181d-5p downregulation (Figure 4B). IL-6 and TNF-α levels in the supernatant of HK-2 cells were measured using ELISA, showing that the IL-6 and TNF-α concentrations decreased as the miR-181d-5p level increased after H/R injury (Figure 4D). These data suggest that miR-181d-5p may play important pathophysiological roles in renal IRI inflammatory responses.

### Bioinformatic Analysis and the Time Course of KLF6 Expression and Localization

**FIGURE 5:**
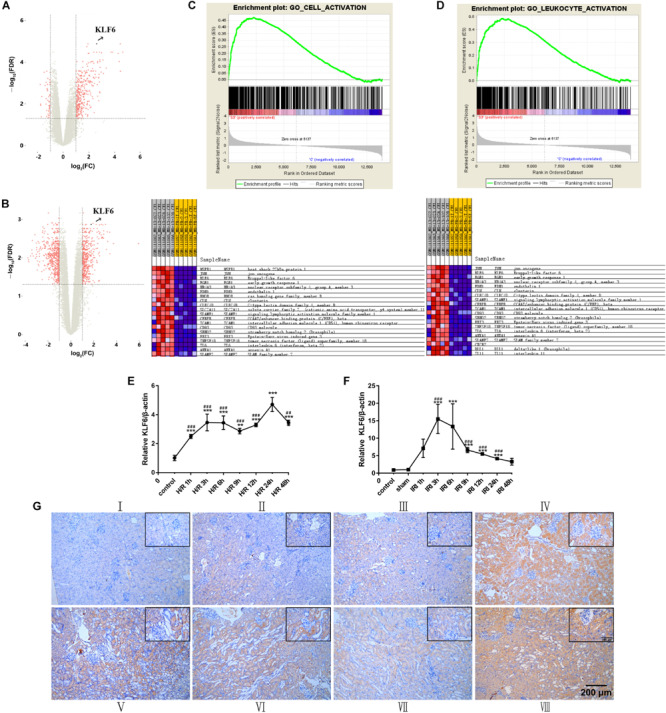
KLF6 expression in IRI and its biological function were predicted by bioinformatic analysis and verified experimentally. **(A,B)** Volcano plot illustrating the differentially expressed proteins identified by quantitative analysis. The –log10 (*P*-value) was plotted against the log2 (IR/Control) ratio. The red dots represent proteins with dysregulated expression in IR samples, and the blue dots indicate KLF6 upregulation in IR samples. **(C,D)** GSEA of microarray data showing enrichment plots and beat maps for sets of genes in the GSE58438 dataset involved in cell activation, leukocyte activation. **(C)** Enrichment plot for the cell activation gene set. Heat map of the core enriched genes in the cell activation gene set. **(D)** Enrichment plot for the leukocyte activation gene set. Heat map of the core enriched genes involved in leukocyte activation. In the enrichment plot, a positive enrichment score (ES; red part of the horizontal bar) indicates gene set enrichment at the top of the ranked list. A negative ES (blue part of the horizontal bar) indicates gene set enrichment at the bottom of the ranked list The middle part of the plot shows the positions of the genes in the gene ranking list. The bottom portion or the plot shows the value of the ranking metric with decreasing rank. Heat map of correlation values for all individual genes within the gene set. The colors and shades indicate the direction and magnitude of correlation. The red boxes indicates a positive correlation with IRI, and the blue boxes indicates a negative correlation with IRI. Darker shades correspond to larger correlation values. The set of genes with the greatest contribution to the ES, i.e., the leading edge subset of genes, is enclosed in a box in both the enrichment plot and the heat map. **(E,F)** Time course of the expression levels of KLF6 in mouse kidneys and HK-2 cells. Tissues were harvested at different time points after the bilateral renal pedicle was clamped for 45 min. *In vitro*, total RNA samples were extracted for RT-PCR, showing significant induction of KLF6 alter hypoxia for 24 h/reoxygenation for 3 h (*n* = *5* per group). **(G)** The localization of KLF6 in the mouse kidney after IRI (45 min of bilateral renal ischemia followed by 24 h of reperfusion) was detected by immunohistochemistry. Positive staining of formalin-fixed, paraffin-embedded mouse kidney sections is indicated by the yellow–brown color. Scale bar, 200 μm. (I: control; II: sham; III: AAV-miR-181d-5p; IV: AAV-shRNA; V: I/R; VI: I/R+AAV-control; VII: I/R+AAV-miR-181d-5p; VIM: IRI+AAV-shRNA.) The data arc presented as the means ± SDs. *P < 0.05, **P < 0.01, ***P < 0.001.

We described the kidney gene expression profiles after treatment with 24 h hypoxia/3 h reoxygenation using volcano plots. The volcano plot of the log2 fold changes versus the −log10 false discovery rates (FDRs) showed transcriptional differences between the I/R group and sham group. The vertical dashed line indicates the cutoff value of a two-fold change in expression, and the horizontal dashed line indicates the FDR cutoff value of 0.05. KLF6 was significantly upregulated in I/R group samples (Figures 5A,B). In addition, gene set enrichment analysis (GSEA) was used to determine biological functions that were significantly altered in acute kidney injury (AKI) blood samples compared to control blood samples. This analysis showed that KLF6 is involved in many crucial pathways related to AKI. GSEA of the GSE58438 gene expression profile indicated that AKI is related mainly to cell activation and leukocyte activation (Figures 5C,D), both of which are closely related to KLF6. In general, these results suggest that KLF6 may be involved in certain cell activation and leukocyte activation processes in AKI. Experimentally, renal IRI was induced in mice via bilateral renal artery occlusion for 45 min and reperfusion for the indicated time. The qRT-PCR results showed that KLF6 levels gradually increased, peaking at 3 h, and that the elevated levels were dramatically reduced 6 h later but remained higher at 24 h than those in the normal and sham groups (Figure 5E). HK-2 cells were cultured for the indicated time under conditions of hypoxia (5% CO_2_, 1% O_2_, and 94% N_2_) followed by 3 h of reoxygenation. Compared with the non-hypoxia-exposed groups, the KLF6 level in the H/R group increased within 12 h but then started to decrease at 24 h (Figure 5F). Interestingly, immunohistochemistry revealed that KLF6 staining was distributed mainly in RTECs in the renal cortex, similar to the pattern observed for miR-181d-5p. Furthermore, the image showed that KLF6 expression was significantly reduced after miR-181d-5p overexpression independent of I/R treatment (Figure 5G), consistent with the previous results (Figure 1B). Collectively, these data suggest that KLF6 may be related to physiological processes of renal IRI.

### KLF6 Aggravates RTEC Damage in the H/R HK-2 Cell Model

**FIGURE 6:**
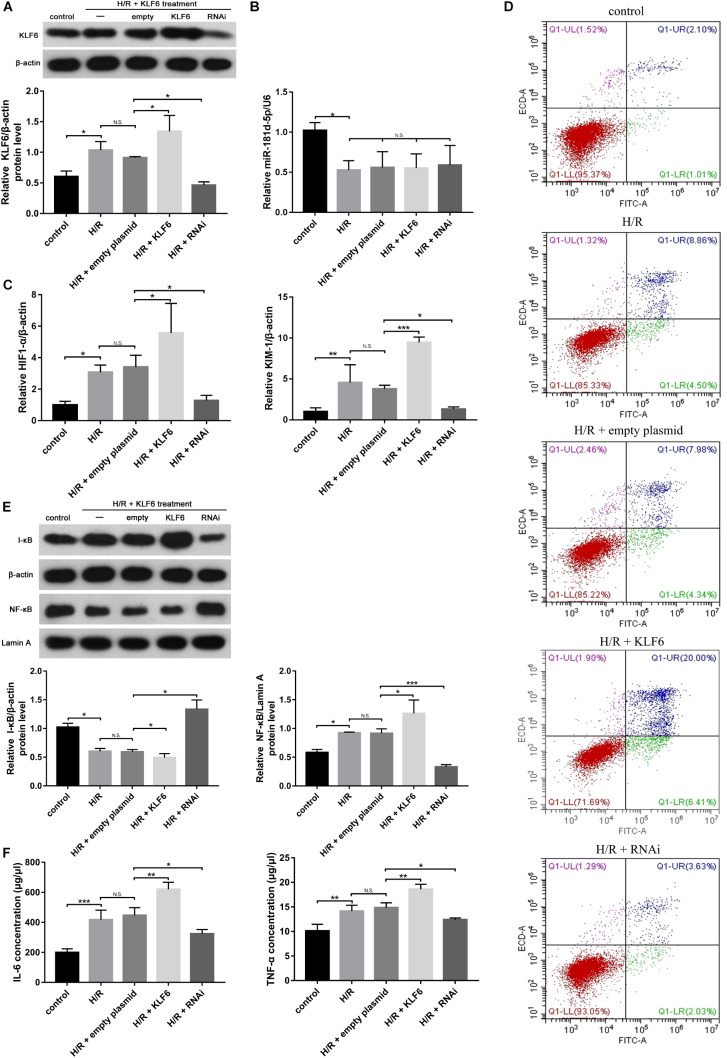
KLF6 overexpression exacerbated the hypoxia-induced decline in renal function, renal tubular cell apoptosis, and inflammatory response. HK-2 cells were transfected with KLF6 plasmid and KLF6 shRNA plasmid or scrambled plasmid with Lipofectamine 3000 and, 72 h later, were incubated in normoxia (control) or treated with hypoxia (1% oxygen) for 24 h/reoxygenation for 3 h. **(A)** KLF6 protein expression in HK-2 cells treated with or without KLF6 (*n* = 3 per group). **(B,C)** qRT-PCR was used to measure miR-181d-5p, KIM-1 and HIF1-α levels after KLF6 transfection (*n* = 5 per group). **(D)** Annexin V-FITC/PI double staining was utilized to evaluate apoptosis after KLF6 transfection. This experiment was repeated three times. **(E)** KLF6 increased NF-KB expression. HK-2 cells were transfected with or without the KLF6 plasmid. The results shown are from Western blot analysis of NF-KB and I-KB.β-Actin and Lamin-A were used as internal controls for I-KB and NF-KB, respectively (*n* = 3 per group). **(F)** ELISAs were used to measure 1L-6 and TNF-α expression levels in the cell supernatant (*n* = 3 per group). The data are presented as the means ± SDs. *P < 0.05, **P < 0.01, ***P < 0.001.

Western blot analysis was conducted to evaluate KLF6 derived from HK-2 cells transfected with empty plasmid, KLF6 plasmid or KLF6-shRNA plasmid and then subjected to control or H/R conditions (Figure 6A). Then, qRT-PCR analysis was used to measure the mRNA levels of miR-181d-5p, KIM-1, and HIF-1α. Interestingly, there was no clear difference between miR-181d-5p levels after transfection with the different plasmids (Figure 6B), but KLF6 plasmid transfection promoted the production of KIM-1 and HIF1-α by RTECs (Figure 6C). Annexin V-FITC/propidium iodide double staining confirmed obvious cell apoptosis after KLF6 transfection (Figure 6D). NF-κB mRNA and protein expression was significantly increased after H/R in HK-2 cells transfected with the KLF6 plasmid, whereas I-κB expression was suppressed specifically by transfection with the KLF6 plasmid (Figure 6E). The ELISA results confirmed that transfection with the KLF6 plasmid attenuated inflammatory cytokine production (Figure 6F). Thus, KLF6 knockdown may be part of a positive feedback mechanism related to apoptosis, the inflammatory response and renal function in renal IRI.

### The 3′UTR of KLF6 Is a Direct Target of miR-181d-5p

**FIGURE 7:**
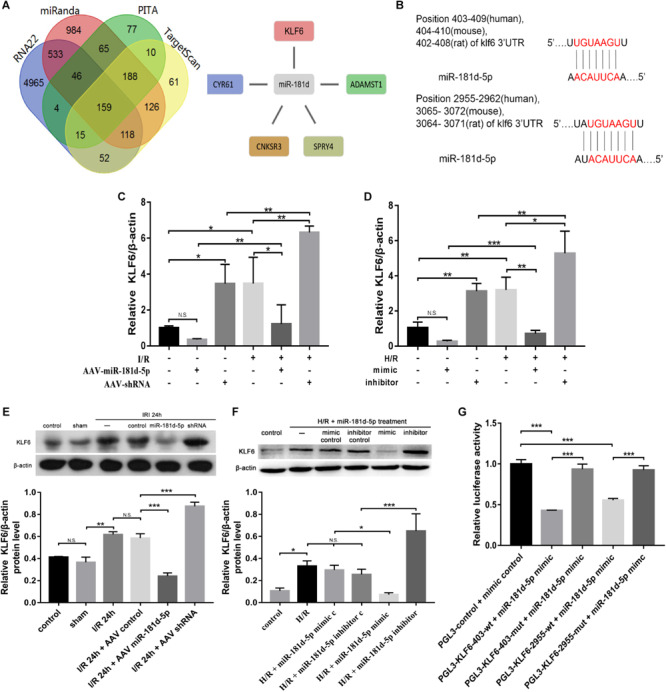
Overexpression of miR-181d-5p inhibited KLF6 expression. **(A)** The four regions indicate the numbers of miRNA-181d target genes obtained via different software packages. The intersecting regions indicate that multiple software packages predicted the same numbers of target genes. The expression of five miRKA-181d target genes (CYR61, CNKSR3, SPRY4, KLF6, ADAMTSI) was increased in AKI (FDR < 0.05, expression level change > 2-fold). **(B)** Schematic representation of the putative miR-181d-5p target sites in the 3’UTR of mouse, rat, and human KLF6. **(C,E)** qRT-PCR and Western blot analyses of KLF6 in mice, mRNA and protein were extracted from kidney cortical tissues for analysis of KLF6, B-Actin was used as the reference gene. For quantification, the KLF6 bands were analyzed by densitometry, and the band densities were expressed as the ratios to β-actin (**C:** *n* = 5 per group; **E:** *n* = 3 per group). **(D,F)** qRT-PCR and Western blot analyses of KLF6 expression in HK-2 cells. Cells were transfected with the miR-181d-5p mimic or miR-181d-5p inhibitor, and the miR-181d-5p mimic control or miR-181d-5p inhibitor control was used as the respective control. Whole-cell lysates were collected for analysis after transfection for 72 h and treatment with hypoxia for 24 h/reoxygenation for 3 h (**D:** *n* = 5 per group; **F:** *n* = 3 per group). **(G)** KLF6 3’UTR activity assay. EGFP-miR-181d-5p and RFP-KLF6 3’UTR plasmids containing fluorescent constructs were co-transfected into 293T cells with or without scrambled or mutant plasmids. The fluorescence intensity was determined 48 h after transfection. The ratio of the normalized sensor fluorescence intensity to the control fluorescence intensity is shown (*n* = 3 per group). The data are presented as the means ± SDs, *P < 0.05, **P < 0.01, ***P < 0.001.

Using RNA22, miRanda, PITA and TargetScan, we determined the number of targeted genes predicted by different systems for miR-181d. Then, we combined the prediction results with the Gene Expression Omnibus (GEO) GSE58438 dataset and found that miRNA-181d had five target genes with increased expression in AKI (FDR < 0.05, expression fold change > 2) (Figure 7A). Using miRbase^1^, we identified that nucleotides 404–410 and 3065–3072 in the 3′UTR of mouse KLF6, nucleotides 402–408 and 3064–3071 in the 3′UTR of rat KLF6, and nucleotides 403–409 and 2955–2962 in the 3′UTR of human KLF6 are complementary to miR-181d-5p seed sequences (Figure 7B). We examined the expression relationship of miR-181d-5p and KLF6 under IRI conditions *in vivo* and *in vitro*. For miR-181d-5p overexpression and inhibition, we used AAV vectors in mice and a miR-181d-5p mimic and inhibitor in HK-2 cells. Experimentally, KLF6 expression was significantly decreased after miR-181d-5p upregulation but increased after miR-181d-5p downregulation in the model of renal ischemia for 45 min/reperfusion for 24 h (Figures 7C,E). In the HK-2 cell model of hypoxia for 24 h/reoxygenation for 3 h, KLF6 expression was suppressed specifically by transfection of the miR-181d-5p mimic, whereas transfection of the miR-181d-5p inhibitor increased KLF6 expression (Figures 7D,F). To study the direct transcriptional interaction between miR-181d-5p and KLF6, the 3′UTR of KLF6 was cloned into the red fluorescent protein C1 (pRFP-C1) vector downstream of the fluorescent reporter gene (RFP-KLF6–3′UTR), and precursors of miR-181d-5p (pre-miR-181d-5p) were cloned into enhanced green fluorescent protein (pEGFP) plasmids (pEGFP-pre-miR-181d-5p). 293T cells were transiently co-transfected with both RFP-KLF6–3′UTR and pEGFP-pre-miR-181d-5p, resulting in significant inhibition of luciferase activity compared with that after transfection of RFP-KLF6–3′UTR alone. The fluorescence intensity was reversed and restored to the control level by miR-181d-5p inhibitor transfection (Figure 7G). These data confirm that miR-181d-5p binds to the KLF6 3′UTR directly and inhibits KLF6 transcription both *in vivo* and *in vitro*.

### MicroRNA-181d-5p Ameliorates Renal IRI by Targeting KLF6

**FIGURE 8:**
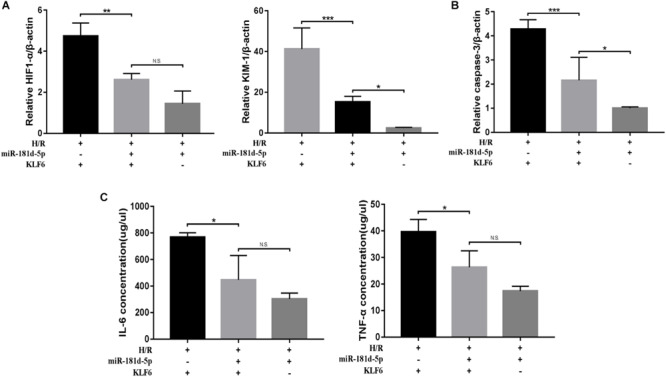
MiR-181d-5p targets KLF6 to ameliorate H/R injury. HK-2 cells were co-transfected with the KLF6 plasmid and miR-181d-5p mimic with Lipofectamine 3000 and, 72 h later, were treated with hypoxia (l% oxygen) for 24 h/reoxygenation for 3 h. **(A,B)** Quantitative analysis of HIF1-α, KIM-1, and caspase-3 expression in HK-2 cells treated with or without miR-181d-5p and KLF6 (*n* = 4 or 5 per group). **(C)** ELISAs were used to measure 1L-6 and TNF-α expression levels in the cell supernatant (*n* = 4 per group). The data are presented as the means ± SDs. ^∗^*P* < 0.05, ^∗∗^*P* < 0.01, ^∗∗∗^*P* < 0.001.

qRT-PCR analysis of KIM-1, HIF-1α, and caspase-3 was conducted on cDNA derived from HK-2 cells transfected with or without the KLF6 plasmid and miR-181d-5p mimic and then subjected to H/R treatment. Compared with the KLF6 overexpression group, the KLF6 and miR-181d-5p co-transfection groups exhibited significantly decreased KIM-1, HIF1-α, and caspase-3 mRNA levels. In addition, the levels of KIM-1, HIF1-α, and caspase-3 were higher in the co-transfection groups than in the miR-181d-5p overexpression group (Figures 8A,B). ELISA showed similar difference for the IL-6 and TNF-α concentrations (Figure 8C). Collectively, these results show that KLF6 co-transfection weakens the protective effect of miR-181d-5p on kidney injury. Thus, KLF6 plays an important role in ameliorating renal IRI as the target of miR-181d-5p.

## Discussion

MiRNAs are well-studied posttranscriptional regulators that have crucial effects on various biological processes by repressing mRNA transcription. An increasing number of studies have demonstrated that miRNAs are involved in the regulation of postischemic pathological injury through multiple pathways. Our findings in this study are the first to identify miR-181d-5p as a significant posttranscriptional regulator in the context of ischemic AKI. MiR-181d-5p was downregulated both *in vivo* in I/R-exposed kidneys and *in vitro* in renal proximal tubular cells cultured under H/R conditions. MiR-181d-5p improves kidney function and reduces apoptosis and the inflammatory response both *in vivo* and *in vitro*. Not only have bioinformatic analyses shown that KLF6 is related to renal IRI, but experiments have also shown that it aggravates renal injury and enhances NF-κB signaling pathway activity, which is involved in IRI-induced inflammation, *in vitro*. Interestingly, miR-181d-5p inhibits KLF6 expression by directly binding the 3′UTR of the KLF6 mRNA, as predicted by several bioinformatics software programs and confirmed by luciferase reporter gene assays. Finally, our collective database analysis results and experimental findings strongly confirm that miR-181d-5p contributes to IRI improvement through targeted inhibition of KLF6.

Our study shows that miR-181d-5p contributes to immune inflammation in AKI through the NF-κB signaling pathway. However, early research on miR-181d focused mainly on the regulation of immune cells. For example, upregulation of miR-181d leads to CD4+CD8+ T cell developmental disorder while also reducing the number of CD4+CD8+ T cells and increasing stress-induced thymic atrophy, but only a slight decrease in peripheral T cells was observed in mice after miR-181d transfection (Belkaya and van Oers, 2014). Dan et al. found that miR-181d overexpression caused a decrease in T cells in the spleen and lymph nodes of mice; additionally, miR-181d can target TNF-α to reduce serum TNF-α levels. Therefore, miR-181d reduces the inflammatory response and mortality in mice (Dan et al., 2015). Furthermore, miR-181d promotes DC maturation by activating the NF-κB pathway via targeting the cylindromatosis gene (Su et al., 2017). Numerous previous studies confirmed that the immune and inflammatory response in AKI involves both the innate and adaptive immune responses. The innate immune response system includes neutrophils, monocytes, dendritic cells, regulatory T cells, natural killer cells, and natural killer T cells, which are activated after AKI and participate in inducing kidney inflammation (Li et al., 2007, 2010; Jang and Rabb, 2009; Martina et al., 2014). The adaptive immune response is activated by specific antigens via processes such as interaction with mature DCs and antigen presentation, T lymphocyte proliferation and activation and T-B lymphocytes. In AKI, the adaptive immune response is initiated slightly later than the innate immune response, but it has important regulatory effects in both the acute injury and repair phases. Therefore, in exploring the effect of miR-181d on AKI and the underlying mechanism, we consider that miR-181d may effectively regulate immune inflammatory cells, a possibility needing further exploration.

KLF6 belongs to the zinc finger transcription factor family and has been reported to shuttle between the cytoplasm and the nucleus (Rodríguez et al., 2010). Our study found that KLF6 overexpression can promote and KLF6 inhibition can inhibit NF-κB activation and the transcription of its downstream genes. Similarly, in intestinal inflammation, KLF6 promotes proinflammatory gene expression by enhancing NF-κB signaling while suppressing anti-inflammatory gene expression by inhibiting STAT3 signaling (Goodman et al., 2016). KLF6 can interact with NF-K B in the nucleus through its transcription activation domain, and this interaction can be enhanced by TNF-α stimulation. Under stimulation by IL-1, KLF6 can be recruited to the promoter of downstream genes in a manner dependent on p65, and in turn, recruited KLF6 promotes the binding of p65 with the promoters of its downstream genes (Zhang et al., 2014). In brief, KLF6 is a coactivator of NF-κB. I-κB, as a negative feedback regulator, binds with NF-κB in the cytoplasm and prevents it from participating in interactions in the resting state. When cells are stimulated by upstream ligands such as TNF-α, I-κB is degraded, and NF-κB is released into the nucleus to regulate downstream genes. Interestingly, a previous study found that KLF6 does not affect the degradation of I-κB or the nuclear translocation of p65, which is different from our results (Figure 6). We believe that unlike in inflammatory bowel disease, in AKI, I-κB degradation may occur, promoting the binding of p65 to its downstream gene promoter (Lan et al., 2012).

In various pathological processes, miR-181d may target various downstream genes. We showed the number of miR-181d target genes predicted by different systems (Figure 7A). Combining these findings with the prediction results for the GSE58438 GEO dataset, we found that miR-181d targeted not only KLF6 but also four other genes with increased expression in AKI: cysteine rich angiogenic inducer 61 (CYR61), CNKSR family member 3 (CNKSR3), sprouty RTK signaling antagonist 4 (SPRY4), and a disintegrin and metalloproteinase with thrombospondin motifs 1 (ADAMST1). However, little is known about the function and regulatory mechanisms of miR-181d-related genes. We summarize the literature and find that miR-181d-5p downstream genes play an important role in inflammation or cell growth related to renal IRI mechanism. ADAMTS1 is a secreted extracellular matrix (ECM) protease that increases in concentration in response to LPS-induced systemic inflammation and is positively regulated by proinflammatory cytokine IL-1β (Ng et al., 2006; Oveland et al., 2012). Chen showed that miR-181d-regulated Adamts1 enzymatically impairs adipogenesis through ECM remodeling (Chen et al., 2016). CNKSR3, which is involved in MAPK pathway regulation, is required for the maintenance of transepithelial sodium transport in the kidney (Ziera et al., 2009). Research on Spry4 has focused predominantly on cancer. For example, SPRY4 affects cell growth as a downstream effector of Wnt7A/Fzd9 signaling in ovarian cancer (Chen et al., 2017), breast cancer (Tian et al., 2018), colorectal cancer (Bienz and Clevers, 2000) and prostate cancer (Bisson and Prowse, 2009). As stated above, miR-181d plays an oncogenic role in breast cancer by repressing SPRY4 (Bisson and Prowse, 2009). What’s more, miR-31 directly targets SPRY4 to mediate the transition from the inflammation to the reepithelialization phases of wound healing (Shi et al., 2018). Previous research by our group shows that CYR61 not only has protective effects on RTECs in the early stage of I/R-AKI, but also attenuates P53/P21/Rb senescence signaling pathway to inhibit fibroblasts proliferation after renal IRI (Liu et al., 2019), though miR-181d-related CYR61 have not yet explored. Thus, we speculate that these miR-181d-related targeted genes may influence I/R-related nephropathy. We aim to establish an early miRNA-kidney relationship network and analyze predictive value of prognosis to provide a reference for prognostic evaluation and targeted therapy of renal IRI patients.

In summary, we showed that miR-181d-5p is downregulated in IRI, which inhibits klf6 and IRI-related factors, ameliorating kidney function and decreasing the apoptosis and inflammatory response caused by I/R. Considering its merits, miR-181d-5p may serve as a therapeutic agent for AKI. Validating its clinical effect and determining more effective approaches for targeted transport to the kidneys are the next steps.

## Materials and Methods

### Animal Treatment to Establish Renal I/R

C57BL/6 male mice (8–10 weeks) were purchased from Charles River Laboratories (China) and housed under specific pathogen-free (SPF) conditions. Mice were anesthetized with intraperitoneal pentobarbital (50 mg/kg) and subjected to bilateral renal artery occlusion for 45 min and reperfusion for the indicated time. The kidneys were observed until the color turned bright red which confirmed reperfusion. The sham operation was identical to the treatment operation, except renal pedicle clamping was not performed. Blood samples were obtained from the orbital sinus while other tissue samples were stored at −80°C until use. All animal experiments were approved by the Medical Ethics Committee at The Affiliated Hospital of Qingdao University, Qingdao (ethics number: QYFYWZLL25661).

### Cell Lines and Cell Culture Treatment

HK-2 cells were purchased from the Shanghai Institute of Cell Biology (CAS, Shanghai, China). The cells were derived from a population from the American Type Culture Collection (ATCC) and were authenticated by short tandem repeat (STR) analysis. HK-2 cells were cultured in DMEM-F12 medium (12400024, Gibco, United States) mixed with 10% fetal bovine serum (16141061, Gibco, United States) and 100X penicillin–streptomycin solution (10 KU/m penicillin, 10 mg/ml streptomycin, P1410, Solarbio, China) and incubated in a 37°C humidified incubator in an atmosphere of 5% CO_2_. For hypoxia treatment, cells were plated to 80% confluence, and the medium was replaced with glucose-free serum-free medium before H/R treatment. H/R group cells were exposed to 24 h of hypoxia (5% CO_2_, 1% O_2_, and 94% N_2_) followed by 3 h of reoxygenation for the experimental time period. Control cells were incubated under normoxic conditions without a medium change.

### *In vitro* Transfection Experiment

MiR-181d-5p mimic or inhibitor or scramble sequence oligos for *in vivo* use was delivered by following the miRNA Product Instructions RN: R10034 from Guangzhou RiboBio. Transfection reagent lipofectamine 3000 was delivered by following the Lipofectamine 3000 Transfection Reagent Instructions from Thermo Fisher. Briefly, mimic and inhibitor working concentrations are, respectively, 50 nM and 150 nM. After the cells were 70% confluent in 6-well plate, 2 ml transfection complex was prepared by mixing miRNA mimic/inhibitor 5/15 ul, lipofectamine3000 5 ul and cell culture medium. The mixture was incubated in a CO_2_ incubator at 37°C for 72 h and then changed the medium for hypoxia/no hypoxia experiments. The KLF6 plasmid was purchased at Genechem Co. in Shanghai, China. HK-2 cells were seeded in a six-well plate (3 × 10^5^ cells / ml per well) and replaced by fresh DMEM-F12 medium with serum until the cells were 70% confluent. Lipofectamine 3000 reagent was diluted in serum-free DMEM-F12 medium (5 ul: 125 ul per well) and plasmid-P3000-serum-free medium complex (5 ug: 10 ul: 250 ul per well) was prepared. Two complex were mixed in a 1:1 ratio, incubated for 15 min at room temperature and then added to HK-2 cell culture dish evenly. The HK-2 cells ware incubated in a CO_2_ incubator at 37°C for 72 h and then changed the medium for hypoxia/no hypoxia experiments. MiR-181d-5p and KLF6 co-transfection was used the same reagent volume and transfection method as described above.

### Renal Parenchymal Injection of AAV

A 100 μl volume of AAV-control, AAV-miR-181d-5p or AAV-shRNA (1 × 10^11^ viral particles, Taitool Bioscience, Shanghai, China) was perfused into both kidneys of mice. Mice were anesthetized with intraperitoneal pentobarbital (50 mg/kg). The renal parenchyma was exposed via a paraspinal incision, and recombinant AAV or phosphate-buffered saline (PBS) was slowly injected via a 30-gauge needle into four sites; subsequently, the needle was removed. The incision was then closed in two layers. Mice were injected penicillin sodium (20000 U per day) intramuscularly for three consecutive days after surgery. All the animals in were free to eat and drink water on the second day after the operation, and the animals’ activities and wounds were observed. There was no obvious abnormality, and the skin tissue of the incision was normal. The mortality rate of each group was 0. Three weeks after gene transfer, mice were anesthetized and subjected to sham or I/R treatment. The kidneys were removed and homogenized for the designated experiments as described previously.

### Target Prediction Analysis

Based on TargetScan predictions, we used three different software programs to further predict the target genes of miR-181d. MiRanda has a wide range of applications to analyze and predict miRNA target genes via sequence matching, double-stranded miRNA and mRNA thermal stability data, and target site conservation. The RNA22 method predicts related miRNAs considering mRNAs but does not consider the conserved species types. PITA software predicts related microRNAs based on target site accessibility. We identified miRNAs that potentially bind the 3′UTR of KLF6 with miRbase (see text footnote 1) and TargetScan^2^ and narrowed the search to miRNAs downregulated in AKI. The selected miRNAs were validated in cisplatin-stimulated HK-2 cells.

### Luciferase Reporter Assay

The promoter sequence of KLF6 was cloned into the pGL vector (E1751, Promega, Madison, WI, United States) upstream of the luciferase sequence. 293T cells were seeded into 96-well plates (10,000 cells per well). After 24 h, 293T cells were co-transfected with the miR-181d mimic or a scrambled miRNA sequence and PGL3-KLF6-wt or PFL3-KLF6-mut using Lipofectamine 3000 transfection reagent (L3000015, Thermo Fisher Scientific, Waltham, MA, United States). Forty-eight hours later, the cells were lysed, and the firefly and Renilla luciferase activities were measured with a Dual-Luciferase Reporter Assay System (Promega, Madison, WI, United States) according to the manufacturer’s protocol.

### Renal Function Assessment

The function of postischemic kidneys was assessed by measuring serum BUN and creatinine levels. For serum preparation, blood was collected in BD Vacutainer serum collection tubes (BD, Franklin Lakes, NJ, United States) and allowed to clot for 1 h at room temperature. After centrifugation at 3000 × *g* for 15 min, the serum supernatant was transferred to a clean tube and immediately stored at 80°C. The samples were evaluated using a Hitachi 7180 automatic biochemical analyzer (Hitachi, Shanghai, China).

### RNA Extraction, Reverse Transcription PCR, and qRT-PCR

Total RNA was extracted using TRIzol (T9424, Sigma, Japan), and cDNA was synthesized using a PrimeScript RT Reagent Kit with gDNA Eraser (RR047A, Takara, Japan) with stem-loop reverse transcription primers. RT-PCR was carried out in an ABI-7500 instrument using TB Green Premix Ex Taq II (RR820A, Takara, Japan). The conditions were as follows: 95°C for 1 min followed by 40 cycles at 95°C for 15 s, 60°C for 15 s, and 72°C for 45 s. Melting curve analysis and sequencing were used to confirm the specificity of the PCR products. The expression of the target gene was normalized to that of the reference gene via the 2−ΔΔCt method. The primers used for qRT-PCR analysis are listed in Table 1.

**TABLE 1:** Primers used.

Target gene	Primer sequence, 5′–3′	
**miR-181d-5p stem loop**	GTCGTATCCAGTGCAGGGTCCGAGGTATTCGCACTGGATACGACACCAC	
	**Forward**	**Reverse**
**miR-181d-5p**	GCAAACATTCATTGTTGTCGGT	CCAGTGCAGGGTCCGAGGT
**h-caspase-3**	AGCAAACCTCAGGGAAACATTC	TGGCTCAGAAGCACACAAACA
**h-HIF1-α**	ACCGCTGAAACGCCAAAG	TCCATCGGAAGGACTAGGTGTCT
**h-KIM-1**	TGGCAGATTCTGTAGCTGGTT	AGAGAACATGAGCCTCTATTCCA
**h-KLF6**	CGGACGCACACAGGAGAAAA	CGGTGTGCTTTCGGAAGTG
**U6**	CGCTTCGGCAGCACATATACTA	GGAACGCTTCACGAATTTGC
**h-β -actin**	GGGAAATCGTGCGTGACATT	GGAACCGCTCATTGCCAAT
**m-KIM-1**	TCCACACATGTACCAACATCAA	GTCACAGTGCCATTCCAGTC
**m-IL-6**	ACCACTCCCAACAGACCTGTCT	CAGATTGTTTTCTGCAAGTGCAT
**m-TNF-α**	ACAAGGCTGCCCCGACTAC	TGGGCTCATACCAGGGTTTG
**m-caspase-3**	GAGGAGATGGCTTGCCAGAA	CTTGTGCGCGTACAGCTTCA
**m-KLF6**	CGGACGCACACAGGAGAAAA	CGGTGTGCTTTCGGAAGTG
**m-β -actin**	ACTGCCGCATCCTCTTCCT	TCAACGTCACACTTCATGATGGA

### Western Blot Analysis

The mice kidneys and HK-2 cells were homogenized in ice-cold standard RIPA buffer with PMSF and phosphatase inhibitor cocktail (Solarbio, Beijing, China) at the ratio of 1000:10:1, followed by centrifugation at 4°C for 15 min at 12,000 × *g*. Protein concentrations were determined by BCA Protein Assay Kit (Solarbio, Beijing, China) and a microplate reader (M5; MD-SpectraMax, Molecular Devices, San Jose, CA, United States). Equal amounts (40 μg/10 μl) of protein were subjected to SDS-PAGE, followed by transfer to polyvinylidene fluoride membranes (PVDF, Millipore, Burlington, MA, United States), that were activated with methanol. After blocking with 5% skimmed milk powder in TBST (pH = 7.6–7.9) for 2 h at room temperature, we used primary antibodies in 3% fetal bovine serum (10099141, Solarbio, Beijing, China) incubated at 4°C overnight [anti-KLF6, 1:2000, 14716-1-AP, Proteintech, United States; anti-NF-κB p65, 1:1000, 8242T, Cell Signaling Technology (CST), United States; anti-I-κB, 1:1,000, 2859, CST, United States; anti-β-actin, 1:10,000, 4970S, CST, United States; anti-IL-6,1:1,000, 12912, CST, United States; anti-TNF-α, 1:1,000, 3707S, CST, United States; anti-lamin A, 1:1,000, GTX101127, GeneTex, United States]. After TBST washing twice, the secondary antibody was goat anti-rabbit IgG-HRP (horse radish peroxidase, 1:5,000, abs20002ss, Absin, Shanghai, China) incubated at room temperature for 1 h. The protein bands were developed by using Immobilon Western Chemiluminescent Substrate (Pierce, United States). The bands were subjected to gray scale analysis using ImageJ software.

### Histopathological Studies

Kidneys from all treated groups were fixed in 10% buffered formalin overnight at 4°C and embedded in paraffin. Sections (100 μm) were stained with HE and analyzed by light microscopy. Renal tubular damage was indicated by the Paller method. Using this system, under 10 high magnification fields of view were taken randomly from 100 renal tubules (Paller and Neumann, 1991). Apoptosis in renal tissues was identified by a TUNEL assay with an In Situ Cell Death Detection Kit (Roche Applied Science, Indianapolis, IN, United States) following the manufacturer’s instructions. TUNEL-positive cells were counted at a magnification of ×400 in 10 randomly selected fields of each slide, and TUNEL-positive cells per field-labeled were quantified as the total kidney cell percentage. Immunohistochemical identification of KLF6 was performed as follows. The paraffin sections were placed in 100, 100, 90, 80, 70% ethanol for 5 min, respectively, and followed by dimethylbenzene dewaxing for 10 min. After washing in 0.01M PBS, the following immunostaining protocols as described as follows: blocking buffer: 1% BSA in 0.01M PBS. Primary antibody: anti-KLF6 antibody (1:2000, 14716-1-AP, Proteintech, United States), incubated overnight at 4°C; HRP secondary antibody (PV-6001; Zsbio, Tianjin, China) for 20 min at 37°C. They were then stained using a DAB Kit (ZLI-9018; Zsbio). Morphology was assessed using a light microscope (CX31; Olympus, Tokyo, Japan). KLF6 area ratio was analyzed using ImageJ software.

### *In situ* Hybridization

Slides were deparaffinized and incubated with 3% H_2_O_2_ for 10 min at 37°C to inactivate endogenous peroxidase and washed with PBS. Proteins were removed from the sections via incubation with 10 mg/ml proteinase-K at 37°C for 10 min, and the sections were then fixed with 1% paraformaldehyde solution containing DEPC (1:1000) at room temperature for 10 min. After washing in PBS, the sections were prehybridized in prehybridization buffer for 4 h at 38°C. Hybridization with ribonucleic probes was then conducted in the dark overnight at 38°C. The hybridization buffer contained bis-DIG LNATM microRNA probe (5 nM) for miRNA-181d-5p or scramble-miRNA as a negative control (Bohai, Hebei, China). After hybridization, the sections were washed in 5× sodium citrate solution (SSC) for 10 min, 0.5 × SSC for 15 min, and 0.2 × SSC at room temperature for 15 min. DAPI was used to stain the specimens in the dark at room temperature for 20 min.

### ELISA

The cell culture supernatants were transferred to sterile centrifuge tubes, centrifuged at 1000 × *g* for 10 min at 4°C, and the supernatants were aliquoted into small EP tubes and stored at −20°C until use. The levels of inflammatory factors in supernatants, including TNF-α and IL-6, were determined by an ELISA system (SEKH-0047, SEKH-0013, Solarbio, China). All experiments were performed according to the manufacturer’s protocols.

### Flow Cytometry

Programmed cell death was evaluated by flow cytometry (BD FACSVerse; BD Biosciences, Franklin Lakes, NJ, United States) using an AV-FITC/PI Apoptosis Detection Kit (Annexin V-FITC and propidium iodide; A005-3; 7 Sea Pharmtech, Shanghai, China). In brief, cells of the four groups were harvested by trypsin digestion and washed twice with PBS. Cells were stained with 5 ml of Annexin V-FITC and 10 ml of PI in the dark. Staining was detected with flow cytometry, and data were analyzed with FlowJo 7.6 software (FlowJo, Ashland, OR, United States).

### Cytoplasmic and Nuclear Protein Extraction

Cell culture preparation and kidney tissue preparation followed the manufacturer’s protocols (Thermo Scientific, 78833, United States). In the process of cytoplasmic and nuclear protein extraction, maintain the volume ratio of CER I:CER II:NER reagents at 200:11:100 μL. According to this protocol, in brief, addition of the first two reagents to a cell pellet made cell membrane disruption and release of cytoplasmic contents. After recovering the intact nuclei from the cytoplasmic extract by centrifugation, the proteins were extracted out of the nuclei with the third reagent.

### Statistical Analyses

Values are expressed as the means ± SDs from at least four experiments. Statistical significance was analyzed using ANOVA followed by the Bonferroni *post hoc* test. A value of *P* < 0.05 was considered statistically significant.

## Data Availability Statement

All datasets generated for this study are included in the article/supplementary material.

## Ethics Statement

This study was carried out in accordance with the recommendations of “International Association of Veterinary Editors guidelines.” The protocol was approved by the “Medical Ethics Committee of the Affiliated Hospital of Qingdao University.”

## Author Contributions

YZ performed the design, experiments, and thesis writing. CG performed the animal experiments and experimental guidance. BZ, LoZ, and WJ performed the design and experimental guidance. CL performed the bioinformatics analysis and data analysis. LW and LiZ performed the animal experiments and data analysis. CY and JD performed the cell experiments and data analysis. YX performed the design, experimental guidance, and data analysis.

## Conflict of Interest

The authors declare that the research was conducted in the absence of any commercial or financial relationships that could be construed as a potential conflict of interest.

## Footnotes

1 http://www.mirbase.org/

2 http://www.targetscan.org/
